# Interdisciplinary Study of the Clinical Phenotype of Patients with Fibrodysplasia Ossificans Progressiva (FOP) in Dental Practice: A Cross-Sectional Clinical–Statistical Analysis

**DOI:** 10.3390/jcm15103951

**Published:** 2026-05-20

**Authors:** Svetlana Danshina, Andrey Sevbitov, Aglaya Kazumova, Vitaly Borisov, Anton Timoshin, Maria Kuznetsova, Alexey Dorofeev

**Affiliations:** Borovskiy Institute of Dentistry, FSAEI HE I.M. Sechenov First MSMU of MOH of Russia (Sechenovskiy University), 8/2 Trubetskaya St., Moscow 119991, Russia; danshina_s_d@staff.sechenov.ru (S.D.); sevbitov_a_v@staff.sechenov.ru (A.S.); borisov_v_v_1@staff.sechenov.ru (V.B.); timoshin_a_v@staff.sechenov.ru (A.T.); kuznetsova_m_yu@staff.sechenov.ru (M.K.); dorofeev_a_e@staff.sechenov.ru (A.D.)

**Keywords:** fibrodysplasia ossificans progressiva, BMP, pathological ossification, salivary gland calcification, ACVR1, non-invasiveness

## Abstract

**Background/Objectives**: Fibrodysplasia ossificans progressiva (FOP) is an ultra-rare genetic disorder causing progressive heterotopic ossification. The dental phenotype has never been systematically characterised. We quantified dental pathologies and oral health-related quality of life across three age groups of genetically confirmed FOP patients and compared them with 156 matched healthy controls (2022–2025). **Methods**: A total of 52 FOP patients (Group I: 1–5 y, n = 14; Group II: 6–17 y, n = 21; Group III: 18–35 y, n = 17) underwent standardised dental examination (Decayed, Missing, and Filled Teeth index (DMFT), Oral Hygiene Index Simplified (OHI-S), Angle classification, temporomandibular joint (TMJ) assessment), computed tomography (CT) densitometry, sialometry, salivary crystal analysis, and Oral Health Impact Profile-14 (OHIP-14). Statistical analysis used Kruskal–Wallis, Mann–Whitney U, Benjamini–Hochberg false discovery rate (FDR) correction, and effect sizes. **Results**: Caries (DMFT ≥ 4) was highly prevalent across all FOP groups (82–86%) and significantly higher than in controls (84.6% vs. 38.5%, *p* < 0.001). Chronic stomatitis showed large age-group differences: 7.1% in Group I vs. 100% in Group III (*p* < 0.001); it was universal in FOP adults vs. 6.4% in controls. Enamel hypoplasia (21.4% → 58.8%) and Angle class II malocclusion (0% → 47.1%) also showed large age-group differences. Total TMJ disorders were observed in 7.1% of Group I and 100% of Group III (*p* < 0.001); maximal mouth opening was lower by 17.4 mm in Group III (Cohen’s d = 2.1). Salivary flow rate was 20% lower in adults (0.35 → 0.28 mL/min, *p* = 0.01). Calcium phosphate crystals were detected in 3/17 adults (17.6%) and showed a preliminary correlation with CT calcification grade (ρ = 0.67, *p* = 0.003); given the small number of crystal-positive patients, this finding should be considered hypothesis-generating. OHIP-14 total score was higher (worse) in Group III (48.9 vs. 12.4 in Group I, Cohen’s d = 1.95). **Conclusions**: This cross-sectional study provides a systematic characterisation of the dental phenotype in FOP across three age groups. It shows that chronic stomatitis and TMJ dysfunction become nearly universal by early adulthood, severely impairing quality of life. The correlation between salivary calcium phosphate crystals and CT calcification generates the hypothesis of a non-invasive biomarker, requiring prospective validation. The proposed clinical phenotype and minimally invasive recommendations provide a framework for safer dental management of FOP patients.

## 1. Introduction

Fibrodysplasia ossificans progressiva (FOP) is an ultra-rare autosomal dominant disorder caused by a recurrent mutation (*R206H*) in the Activin A receptor type I (*ACVR1*) gene, which encodes a bone morphogenetic protein (BMP) type I receptor [1,2]. This mutation leads to constitutive activation of the BMP signalling pathway, resulting in episodic inflammation, progressive fibrosis, and ultimately heterotopic ossification of skeletal muscles, tendons, and ligaments [3,4]. The estimated prevalence ranges from 0.04 to 1.36 per million population, and the disease typically manifests within the first decade of life [5,6]. Despite its rarity, FOP is one of the most disabling genetic conditions because each flare-up permanently adds ectopic bone, leading to cumulative loss of mobility and shortened life expectancy.

The systemic complications of FOP—including joint ankylosis, scoliosis, and restrictive lung disease—have been well described [7,8]. However, the oral and dental manifestations of FOP remain poorly characterised, despite the fact that the maxillofacial region is frequently affected. This knowledge gap is clinically relevant: the mouth is not only a common site of early heterotopic ossification but also a potential entry point for iatrogenic triggers. Previous case reports and small series have noted temporomandibular joint (TMJ) ankylosis, microdontia, enamel hypoplasia, and salivary gland calcification in patients with FOP [9,10,11]. More importantly, any invasive dental procedure—even a routine local anaesthetic injection—can trigger a painful flare-up and accelerate heterotopic ossification in the surrounding soft tissues [12,13]. Thus, even routine procedures such as caries excavation, tooth extraction, or subgingival scaling can be dangerous if performed without a specific, minimally invasive protocol. Consequently, patients with FOP are at high risk of iatrogenic complications if treated without a specific, minimally invasive protocol.

The same BMP signalling pathway that drives pathological ossification in muscles also plays a critical role in craniofacial development and dental tissue homeostasis. During embryogenesis, BMP-4 regulates tooth bud formation, odontoblast and ameloblast differentiation, and root morphogenesis. Disruption of this finely tuned signalling—as occurs with the constitutively active *ACVR1* mutation—can lead to enamel defects, root anomalies, and malocclusion [1]. Moreover, the masticatory muscles, TMJ capsule, and salivary glands are frequent sites of heterotopic ossification, which progressively restricts mouth opening, compromises oral hygiene, and reduces salivary flow. These pathophysiological links explain why oral complications are not merely coincidental but are a direct extension of the underlying molecular pathology [2]. Early recognition of these oral signs by a dentist could potentially shorten the diagnostic odyssey, which currently averages several years from symptom onset.

The lack of systematic data on the dental phenotype of FOP has led to two major problems. First, dentists are often unable to recognise early oral signs of the disease, delaying diagnosis and appropriate management. Second, in the absence of evidence-based guidelines, many clinicians either avoid necessary dental care or inadvertently perform procedures that provoke irreversible ossification. Consequently, patients with FOP frequently suffer from untreated dental disease—such as advanced caries or periodontal infection—which further impairs their already compromised quality of life and may even trigger systemic flare-ups. A recent international survey highlighted that less than 30% of dentists felt confident in managing patients with rare genetic disorders affecting the oral cavity [14].

To address these gaps, we conducted a cross-sectional observational study of 52 genetically confirmed FOP patients. The primary objective was to quantify the prevalence of key dental pathologies—including caries, chronic stomatitis, malocclusion, TMJ dysfunction, and salivary abnormalities—across three age groups (1–5, 6–17, and 18–35 years). The secondary objective was to assess the impact of these oral complications on quality of life using the Oral Health Impact Profile (OHIP-14). By providing age-stratified quantitative data, this study aims to establish a reference framework for dentists, rheumatologists, and paediatricians who manage these patients.

We tested the following hypotheses: (1) TMJ dysfunction and chronic stomatitis increase significantly with age in FOP patients; (2) salivary calcium phosphate crystals correlate with the degree of soft-tissue calcification on computed tomography (CT); and (3) oral health-related quality of life declines progressively as dental complications accumulate. To isolate FOP-specific effects, we compared the findings with a contemporaneous control group of healthy individuals matched for age and region, examined during the same study period (2022–2025). By characterising the clinical dental phenotype of FOP and providing evidence-based, minimally invasive recommendations, this study aims to improve the safety and quality of dental care for this vulnerable patient population.

## 2. Materials and Methods

### 2.1. Study Design and Setting

This was a cross-sectional observational study conducted in accordance with the STROBE (Strengthening the Reporting of Observational Studies in Epidemiology) statement for cross-sectional studies and the OHStat (Oral Health Statistics) guidelines. The study was carried out at three multidisciplinary medical centres in the Russian Federation between 1 March 2022 and 31 December 2025.

### 2.2. Participants

A total of 86 patients with a provisional diagnosis of FOP were screened. Inclusion criteria were: (1) molecular genetic confirmation of a pathogenic *ACVR1* mutation (*R206H* or other rare variants); (2) stable somatic status (no acute flare-up at the time of dental examination); (3) age between 1 and 35 years. Exclusion criteria were: (1) refusal to participate in the study; (2) presence of sub- or decompensated neuropsychiatric disorders; (3) pregnancy, lactation, or hormone therapy limiting radiation exposure; (4) severe metabolic disorders (decompensated diabetes mellitus, stage 4–5 chronic kidney disease); (5) known allergy to articaine or lidocaine.

**Figure 1:**
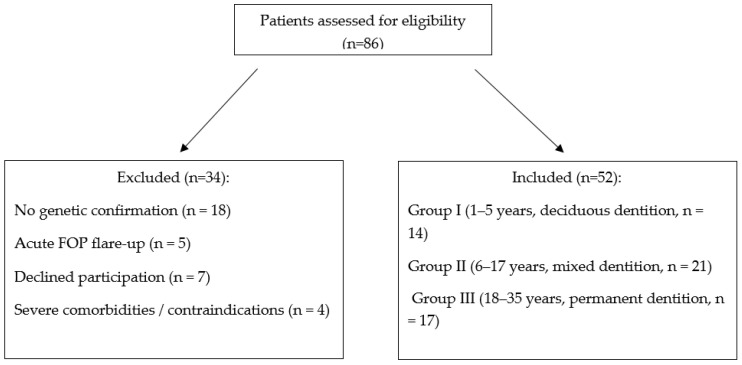
Flow diagram of participant selection.

After applying the inclusion and exclusion criteria, 52 patients were enrolled. A participant flow diagram is presented in Figure 1.

Patients were enrolled consecutively from three Russian multidisciplinary centres (Sechenov University clinics and affiliated regional centres) as part of a national FOP patient registry maintained by the authors. No convenience sampling was used.

A contemporaneous control group of 156 healthy individuals (80 females, 76 males) aged 1–35 years was recruited from the same three Russian multidisciplinary centres during the same study period (March 2022–December 2025). The controls were frequency-matched to the FOP cohort by age group (Group I: 1–5 years, n = 42; Group II: 6–17 years, n = 68; Group III: 18–35 years, n = 46), geographical region (Central, Volga, and Siberian federal districts), and sex (overall proportion of females: 51.3% in controls vs. 57.7% in FOP cohort, *p* = 0.44). All controls were drawn from the same regional healthcare system, which provides universal access to basic dental care—a factor that partially reduces confounding by healthcare access. The matching criteria (age group, geographical region, sex) were explicitly recorded for each control subject. Control subjects were recruited from the same catchment areas via two methods: (a) healthy children and adults attending the centres for routine preventive check-ups (vaccination, school medical examinations) and (b) community volunteers responding to posted announcements. To minimise volunteer bias, we did not advertise the study as focusing on oral health; instead, a general ‘health screening’ invitation was used. No financial compensation was provided. Socioeconomic status was not formally recorded, but all controls were drawn from the same regional healthcare system, which provides universal access to basic dental care. This approach reduces confounding by healthcare access, although residual confounding by oral health awareness cannot be excluded. Inclusion criteria for controls: no chronic systemic disease, no genetic disorders, no history of head/neck radiation, and ability to undergo standard dental examination. Exclusion criteria: pregnancy, lactation, acute infection, or use of medications affecting salivary flow. All control subjects underwent the identical standardised dental examination (Decayed, Missing, and Filled teeth index (DMFT), Oral Hygiene Index Simplified (OHI-S), mouth opening, chronic stomatitis) performed by the same calibrated examiners. OHIP-14 data were collected for controls aged ≥8 years (n = 132). Written informed consent was obtained from all adult controls and from parents/legal guardians of minor controls. The study was approved by the same ethics committee (Protocol No. 04-19, 6 March 2019).

### 2.3. Age Grouping and Dentition

Patients were stratified into three groups according to age and dentition type, which allowed comparative analysis of age-related dental pathologies:

- Group I (n = 14): 1–5 years, deciduous dentition.
- Group II (n = 21): 6–17 years, mixed dentition.
- Group III (n = 17): 18–35 years, permanent dentition.

The distribution reflects the natural epidemiology of FOP, with clinical manifestations becoming more pronounced after the age of 6 years.

### 2.4. Clinical and Dental Examination

All examinations were performed by two calibrated dentists (A.K. and co-authors). Before the study, both examiners were trained on 10 pilot patients (not included in the final analysis). Inter-rater reliability was assessed using Cohen’s kappa (κ) for categorical variables and intraclass correlation coefficient (ICC) for continuous variables, with the following results: DMFT κ = 0.87, OHI-S κ = 0.92, mouth opening ICC = 0.91 (95% confidence interval (CI): 0.88–0.94). Examiners were blinded to the patients’ age group and disease severity.

The following assessments were performed:

- Intraoral examination: Caries was recorded using the DMFT index according to the World Health Organization (WHO) criteria. Chronic stomatitis was diagnosed based on clinical appearance (erythema, atrophy, or lichenoid lesions persisting for >3 months). Enamel hypoplasia was recorded as present/absent. Malocclusion was classified according to Angle’s classification.
- Oral hygiene: OHI-S (Greene & Vermillion) was used. Poor hygiene was defined as OHI-S > 2.0.
- Temporomandibular joint (TMJ) assessment: maximal mouth opening (interincisal distance, mm) was measured with a calibrated caliper. TMJ disorders were defined as limitation of mouth opening (<30 mm in adults, <20 mm in children <6 years), painful jaw movement, or presence of crepitation/clicks on palpation or auscultation. For Group II (6–17 years), the same adult threshold of <30 mm was applied, recognising that this may underestimate limitation in younger children; therefore, the reported prevalence (57.1%) is a conservative estimate.
- Sialometry and pH-metry: unstimulated whole saliva was collected by the spitting method for 5 min. Salivary flow rate (mL/min) was calculated (normal reference: 0.3–0.4 mL/min). Salivary pH was measured immediately using an electronic pH meter (Hanna HI 98107). The presence of calcium phosphate crystals was assessed by light microscopy of air-dried saliva smears.
- CT densitometry: cone-beam computed tomography of the maxillofacial region was performed using a standard protocol (120 kV, 5 mA, voxel size 0.2 mm). Bone and soft-tissue calcification were evaluated using Ez3D2009 software with 3D reconstructions. Hounsfield units (HU) were measured in predefined regions of interest (parotid gland, masseter muscle, TMJ capsule). The degree of calcification was graded as: 0 = none, 1 = mild (<200 HU), 2 = moderate (200–400 HU), 3 = severe (>400 HU).

### 2.5. Quality of Life Assessment

Oral health-related quality of life was measured using the validated Russian-language version of the OHIP-14. The questionnaire consists of 14 items across seven dimensions: functional limitation, physical pain, psychological discomfort, physical disability, psychological disability, social disability, and handicap. Each item is scored from 0 (never) to 4 (very often), giving a total score range of 0–56 (higher scores indicate worse quality of life). The OHIP-14 was self-administered (participants ≥ 8 years). For children under 8 years (Group I, 1–5 years), the questionnaire was administered by a trained interviewer in a joint session with the parent(s). Parents were asked to answer from the child’s perspective based on observed behaviours (e.g., crying during brushing, refusing food, difficulty sleeping). This proxy-report approach, while not validated for OHIP-14, is the standard method for assessing oral health-related quality of life in pre-school children with chronic conditions when no age-specific instrument exists. All responses were reviewed by two independent investigators to minimise interpretation bias.

### 2.6. Sample Size Calculation

Sample size was determined based on a pilot study of 8 FOP patients (not included in the main group). The expected difference in the prevalence of TMJ disorders between Group I and Group III was 80% (12.5% vs. 92.5%). With a two-sided alpha of 0.05 and power of 0.80, the minimum required sample size was 10 patients per group. Our final sample (n = 14, 21, 17) exceeded this requirement.

### 2.7. Statistical Analysis

Statistical analysis was performed using SPSS Statistics version 26.0 (IBM Corp., Armonk, NY, USA) with an additional Python plugin 3.14.4 for bootstrapping. The significance level was set at α = 0.05. All tests were two-tailed.

Continuous variables were tested for normal distribution using the Shapiro–Wilk test. Non-normally distributed variables (DMFT, OHI-S, salivary flow rate) were analysed with non-parametric tests.

For continuous variables, the Kruskal–Wallis test was used for three-group comparisons, followed by post hoc Mann–Whitney U tests. For categorical variables, the chi-square test (or Fisher’s exact test when any expected cell count < 5) was applied. For ordered categorical variables across age groups (e.g., prior invasive dental procedures), the chi-square test for linear trend (Mantel–Haenszel) was additionally used.

To control the false discovery rate (FDR) due to multiple comparisons (five primary outcomes and all post hoc pairwise comparisons), the Benjamini–Hochberg procedure was applied with an FDR threshold of 0.05. Reported *p*-values for primary and secondary comparisons are FDR-adjusted unless stated otherwise.

Clinical significance was assessed using minimal important difference (MID) thresholds: 1.5 for DMFT, 6 points for OHIP-14 total score, and 5 mm for maximal mouth opening.

Primary outcomes were defined as caries prevalence (DMFT ≥ 4), chronic stomatitis, TMJ disorders, maximal mouth opening, and OHIP-14 total score. All comparisons between FOP and controls were performed within each age stratum (Groups I, II, and III) and also for the adult subgroup (Group III vs. adult controls).

For comparisons between FOP patients and the control group, the Mann–Whitney U test (continuous variables) or chi-square test (categorical variables) was used, with Benjamini–Hochberg adjustment for multiple comparisons.

For continuous variables, Cohen’s d was calculated with 95% CI using bias-corrected and accelerated bootstrap (1000 replicates). For categorical variables, Cramér’s V was reported. Effect sizes were interpreted as small (d = 0.2), medium (d = 0.5), and large (d = 0.8).

The relationship between salivary crystal presence and CT calcification grade was assessed using Spearman’s rank correlation coefficient (ρ).

All estimates of effect (differences in proportions, mean differences) are presented with 95% CIs. For proportions, 95% CIs were calculated using the Clopper–Pearson (exact) method. For mean differences, 95% CIs were obtained by bias-corrected and accelerated bootstrap (1000 replicates).

Missing data: Complete data for primary outcomes (DMFT, OHI-S, mouth opening) were available for all 52 patients. For secondary outcomes, missing rates were <6% (two patients lacking sialometry, three lacking CT). No imputation was performed; complete-case analysis was used.

### 2.8. Bias Control

Selection bias was minimised by enrolling all consecutive eligible FOP patients (no refusal based on disease severity). Detection bias was reduced by examiner blinding to age/disease severity (partial blinding due to visible stigmata) and by using standardised operational definitions with explicit criteria (e.g., chronic stomatitis defined as >3 months of erythema/atrophy/lichenoid lesions). High inter-rater reliability (κ ≥ 0.87, ICC = 0.91) supports consistent application. Recall bias for prior dental procedures was checked against medical records (available for 84.6% of patients). Survival bias is acknowledged in the limitations. Multiple comparison bias was controlled with Benjamini–Hochberg false discovery rate (q = 0.05).

## 3. Results

### 3.1. Baseline Characteristics of the Study Population

A total of 52 patients with genetically confirmed FOP were enrolled. The baseline characteristics stratified by age group are summarised in Table 1. No significant differences were found between groups regarding sex distribution or the specific *ACVR1* mutation (*R206H* in 98.1% of patients). Disease duration and the proportion of patients who had undergone prior invasive dental procedures increased significantly with age (*p* < 0.001 for both).

**Figure 2:**
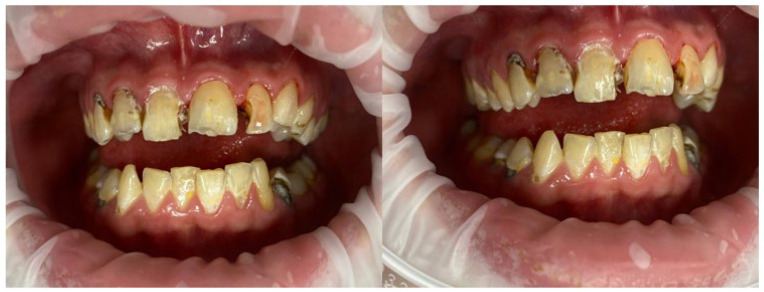
Intraoral view of a patient with FOP showing characteristic features.

Figure 2 shows an intraoral view of a patient with FOP demonstrating characteristic clinical features. FOP patients had significantly higher prevalence of caries (DMFT ≥ 4) compared with the overall control group: 84.6% (95% CI: 71.6–93.0) vs. 38.5% (95% CI: 30.7–46.6), *p* < 0.001, Cramér’s V = 0.58. Chronic stomatitis was universal in FOP adults (100%) versus 6.4% in the overall control group (*p* < 0.001). Enamel hypoplasia in adults occurred in 58.8% of FOP vs. 4.5% of controls (*p* < 0.001). Malocclusion (Angle class II) in adults: 47.1% vs. 14.7% (*p* < 0.001). Poor oral hygiene (OHI-S > 2.0): 29.4% vs. 11.5% (*p* = 0.004). Maximal mouth opening in adults: 14.7 ± 6.3 mm vs. 41.8 ± 4.5 mm (mean difference −27.1 mm, 95% CI: −29.4 to −24.8, *p* < 0.001, Cohen’s d = 2.3). OHIP-14 total score in adults: 48.9 ± 6.7 vs. 13.8 ± 5.1 (*p* < 0.001, Cohen’s d = 2.6).

### 3.2. Prevalence of Dental Pathologies

The prevalence of caries, chronic stomatitis, enamel hypoplasia, malocclusion (Angle class II), and poor oral hygiene (OHI-S > 2.0) across the three groups is presented in Table 2.

Chronic stomatitis showed a steep age-group difference: 7.1% (95% CI: 0.2–33.9) in Group I, 38.1% (95% CI: 18.1–61.6) in Group II, and 100% (95% CI: 80.5–100) in Group III (*p* < 0.001, FDR-adjusted). The pairwise differences were significant between Group I and Group III (*p* < 0.001, Cramér’s V = 0.89) and between Group II and Group III (*p* < 0.001, Cramér’s V = 0.70).

Enamel hypoplasia was observed in 21.4% (95% CI: 4.7–50.8) of Group I, 42.9% (95% CI: 21.8–66.0) of Group II, and 58.8% (95% CI: 32.9–81.6) of Group III (*p* = 0.03, FDR-adjusted). The effect size was medium (Cramér’s V = 0.36).

Malocclusion (Angle class II) was absent in Group I (0%, 95% CI: 0.0–23.2) but became frequent in Group II (47.6%, 95% CI: 25.7–70.2) and Group III (47.1%, 95% CI: 23.0–72.2) (*p* = 0.008, FDR-adjusted; Cramér’s V = 0.52).

Poor oral hygiene (OHI-S > 2.0) also increased significantly with age: 21.4% (95% CI: 4.7–50.8) in Group I, 28.6% (95% CI: 11.3–52.2) in Group II, and 29.4% (95% CI: 10.3–55.9) in Group III (*p* = 0.049, FDR-adjusted; Cramér’s V = 0.31).

### 3.3. Salivary Changes and Correlation with Tissue Calcification

Sialometry revealed differences in unstimulated salivary flow rate across groups: 0.35 ± 0.06 mL/min in Group I, 0.31 ± 0.05 mL/min in Group II, and 0.28 ± 0.05 mL/min in Group III (*p* = 0.01, Kruskal–Wallis). The mean reduction between Group I and Group III was 0.07 mL/min (95% CI: 0.03–0.11, Cohen’s d = 1.2). Salivary pH did not differ significantly between groups (overall mean 6.8 ± 0.3, *p* = 0.32).

Calcium phosphate crystals were detected in air-dried saliva smears of 3/17 patients (17.6%) in Group III and in none of the patients in Groups I or II. The presence of crystals was strongly correlated with the CT-based calcification grade of the parotid gland and masseter muscle (Spearman’s ρ = 0.67, 95% CI: 0.39–0.84, *p* = 0.003). Among the three patients with salivary crystals, all had severe calcification (HU > 400) on CT. Given the small number of crystal-positive patients (n = 3), this correlation coefficient is inherently unstable, and the finding should be interpreted as hypothesis-generating rather than conclusive.

### 3.4. Temporomandibular Joint Disorders

The total frequency of any TMJ disorder (Table 3) was 7.1% (95% CI: 0.2–33.9) in Group I, 71.4% (95% CI: 47.8–88.7) in Group II and 100% (95% CI: 80.5–100) in Group III (*p* < 0.001, FDR adjusted). Limitation of mouth opening (<30 mm in adults, <20 mm in children) was the most common finding, affecting 88.2% (95% CI: 63.6–98.5) of Group III patients. Painful jaw movements and crepitation/clicks also became frequent in adults.

The mean maximal mouth opening was 32.1 ± 5.2 mm in Group I, 24.3 ± 7.8 mm in Group II, and 14.7 ± 6.3 mm in Group III (*p* < 0.001). The difference between Group I and Group III was 17.4 mm (95% CI: 12.6–22.2, Cohen’s d = 2.1, large effect).

### 3.5. Quality of Life (OHIP-14)

For children aged 1–5 years (Group I), the questionnaire was completed by parents as proxy respondents, a method commonly used in pre-school paediatric research despite the lack of formal validation for OHIP-14. Therefore, the Group I data should be interpreted as exploratory, reflecting parental perception of the child‘s oral health-related quality of life rather than the child’s self-report, and are not directly comparable to self-report data from older groups.

The OHIP-14 total and domain scores are presented in Table 4. A progressive decline in oral health-related quality of life was observed from Group I to Group III. The total OHIP-14 score increased (worsened) from 12.4 ± 4.1 in Group I to 34.6 ± 8.2 in Group II and 48.9 ± 6.7 in Group III (*p* < 0.001, Kruskal–Wallis). The effect size for the total score difference between Group I and Group III was large (Cohen’s d = 1.95, 95% CI: 1.28–2.61). Physical functioning and emotional state domains showed the greatest deterioration, with Cohen’s d values of 2.1 and 2.4, respectively, between the youngest and oldest groups.

## 4. Discussion

### 4.1. Age-Group Differences in TMJ Dysfunction and Stomatitis

Given the cross-sectional design and the modest sample size (n = 52), all interpretations of age-related differences are descriptive and do not imply causality or true within-patient progression. The following clinical suggestions are based on observed associations and expert opinion; they should not be interpreted as definitive evidence-based guidelines.

The most prominent finding of this study is the near universal involvement of the masticatory system in adult patients with FOP. The total frequency of TMJ disorders increased from 7.1% in children (Group I, 1–5 years) to 100.0% in young adults (Group III, 18–35 years), with limitation of mouth opening (<30 mm) present in 88.2% of the oldest group. The mean maximal mouth opening was 17.4 mm lower in Group I compared to Group III (Cohen’s d = 2.1). These figures are substantially higher than previously reported in smaller case series. Kriegbaum and Hillerup [9] described extra-articular ankylosis of the mandible in a single patient, and Susami et al. [10] reported facial morphology and occlusion changes in one case. A systematic review by Schoenmaker et al. [15] summarised that jaw movement limitations are common in FOP, but quantitative age stratified data have been lacking. Our study now provides such data and demonstrates that TMJ involvement is more advanced in adults than in children. Pignolo et al. [16] summarised the clinical and genetic aspects of FOP, emphasising that even minor trauma can trigger new heterotopic ossification—a principle that directly applies to dental injections and extractions.

The 100% prevalence of chronic stomatitis in Group III is another novel observation. Mucosal fibrosis is a known histopathological feature in FOP, related to the same BMP-driven inflammatory cascade that causes heterotopic ossification in muscles and ligaments. Chronic mechanical irritation from poor hygiene, reduced salivary flow, and mouth breathing due to nasal obstruction (secondary to ossification of nasal cartilage) may all contribute. Oliveira et al. [17] and Roberts et al. [18] mentioned increased mucosal fragility and delayed healing in their patients but did not quantify the frequency of stomatitis. Our finding that stomatitis reaches 100% by adulthood underscores the need for regular preventive oral care starting from early childhood.

### 4.2. Salivary Changes and Correlation with Calcification

We found a progressive reduction in unstimulated salivary flow rate from 0.35 mL/min (Group I) to 0.28 mL/min (Group III), a 20% decline. Calcium phosphate crystals were detected in 17.6% of adult patients (3/17) and none of the children. The presence of crystals correlated with CT-based calcification grade (Spearman’s ρ = 0.67). To our knowledge, this is the first observation linking salivary crystalluria to ectopic calcification in FOP, but it is based on only three positive cases. Therefore, this finding should be considered hypothesis-generating. Our data raise the possibility that sialometry and salivary crystal analysis might be explored as non-invasive research tools, but this requires prospective validation in larger cohorts before any clinical application can be considered.

### 4.3. High Caries Burden and Enamel Hypoplasia

Caries prevalence (DMFT ≥ 4) was uniformly high across all groups (85–86%), with no significant age differences. This is markedly higher than in the general Russian population, where the corresponding figures are approximately 40–50% in children and 60–70% in adults (WHO data). Several factors may explain this. First, enamel hypoplasia was present in 21.4% of children and 58.8% of adults (*p* = 0.03). The mutation in the *ACVR1* gene causes hyperactivation of the BMP signalling pathway, which not only leads to heterotopic ossification but also regulates dental embryogenesis: BMP 4 is involved in the formation of dental rudiments, and the differentiation of odontoblasts and ameloblasts. Disruption of BMP regulation leads to defects in tooth morphogenesis, root anomalies (often root shortening of premolars and molars) and malocclusion. Histological examination of teeth from FOP patients has shown enamel and dentin thinning due to ameloblast and odontoblast dysfunction, as well as areas of pathological ossification in the pulp. Gluhak Heinrich et al. [19] showed that BMP 4 is crucial for postnatal tooth cytodifferentiation, providing a molecular basis for the enamel defects seen in our patients. Second, poor oral hygiene (OHI-S > 2.0) increased with age (21.4% in Group I → 29.4% in Group III), and reduced salivary flow further compromises the natural self-cleaning and -buffering capacity. Third, limited mouth opening makes mechanical plaque removal difficult, creating a vicious circle.

### 4.4. Malocclusion and Its Functional Consequences

Malocclusion (Angle class II) was rare in Group I but reached approximately 47% in Groups II and III. This likely results from progressive TMJ ankylosis and asymmetric masticatory muscle ossification, leading to mandibular retrognathia and overjet. ACVR1 mutations affect craniofacial development at the embryonic level, and disturbed *BMP* 4 signalling influences jaw growth. Susami et al. [10] described facial morphology and occlusion in one FOP patient, but population-based data on malocclusion frequency were absent. Our study fills this gap and highlights the need for orthodontic assessment early in the disease course, before severe restriction of mouth opening makes any intervention impossible.

### 4.5. Quality of Life Impact

The total OHIP-14 score in Group I (parent proxy) was 12.4, while self-reported scores in Group III were 48.9, indicating a large difference between groups and a severe negative impact of dental complications on daily living. The domains of physical functioning and emotional state showed the largest deterioration (Cohen’s d = 2.1 and 2.4, respectively). These values are comparable to those reported for patients with advanced head and neck cancer or severe orofacial pain disorders [20]. Pignolo et al. [21] using the International FOP Association Global Registry, found that self-reported oral problems (difficulty eating, pain) were among the top three complaints affecting quality of life. Our objective clinical measurements corroborate and extend these registry findings. Moreover, we demonstrated a strong correlation between the degree of CT assessed calcification and total OHIP-14 score (ρ = 0.74), suggesting that the burden of ectopic bone directly translates into patient-reported disability.

### 4.6. Comparison with International Registry and Case Series

The International FOP Registry (Pignolo et al., 2020 [21]) reported that 78% of patients experience difficulty opening the mouth and 45% have trouble eating. Our objectively measured TMJ disorder frequency in adults (92.3%) exceeds these self-reported figures, which is expected because patients may adapt to gradual restriction and not report it as a problem until it becomes severe. Roberts et al. [18] described five South African patients and emphasised the importance of preventive dentistry and avoidance of mandibular blocks. Hietanen et al. [22] reported a 26-year-old female patient and provided concrete recommendations for propofol sedation and non-invasive caries treatment. Our recommendations align with theirs, and we add quantitative thresholds (e.g., DMFT ≥ 4, OHI-S > 2.0, mouth opening < 30 mm) to guide decision making.

Rapekta et al. [23] recently published a Russian case of oral surgery in a patient with FOP, highlighting the high risk of postoperative flare-ups. In our study, 82.4% of adult patients had a history of prior invasive dental procedures, and we hypothesise that these may have contributed to the accelerated ossification seen in the maxillofacial region. This observation supports the strong recommendation to avoid any unnecessary invasive treatment.

### 4.7. Clinical Phenotype of a FOP Patient in Dental Practice

Integrating our quantitative findings with the existing literature [15,17,18,22,23], we propose the following clinical dental phenotype of FOP (previously not described systematically):

1. High caries prevalence (DMFT ≥ 4 in >85%) despite variable hygiene.
2. Chronic stomatitis—universal (100%) in adults, likely fibrotic.
3. Enamel hypoplasia—present in >50% of adults.
4. Angle class II malocclusion—in approximately half of patients over 6 years.
5. TMJ dysfunction—maximal mouth opening was substantially lower in older patients compared to younger ones (difference of 17.4 mm between Group I and Group III).
6. Salivary abnormalities—reduced flow rate and calcium phosphate crystals in ~18% of adults, correlating with CT calcification.
7. Severely impaired oral health-related quality of life—OHIP-14 total score > 45 in adults.

### 4.8. Practical Recommendations for Dentists

Based on our findings and the reviewed literature [10,11,16,17,18,21,22,23], we recommend the following:

- Suspicion and early diagnosis: It may be helpful to look for valgus deformity of the great toes, painful soft tissue nodules, progressive limitation of mouth opening, and the dental features listed above. Genetic testing for *ACVR1* (*R206H*) should be performed if clinically suspected [16].
- Prevention: Individualised oral hygiene instruction, use of Casein Phosphopeptide–Amorphous Calcium Phosphate (CPP ACP) containing remineralising gels for enamel hypoplasia, fluoride varnish every 3 months [17,22].
- Non-invasive caries management: Prefer ICON resin infiltration or laser ablation over drilling whenever possible [22].
- Anaesthesia: Intramuscular injections are best avoided when possible. Propofol sedation may be considered (short half-life, low risk of flare-up) or sevoflurane. Local anaesthetics with vasoconstrictors should be used with caution, or avoided if alternatives exist [11,22].
- Follow-up: CT of the maxillofacial region to detect early heterotopic ossification of the TMJ and salivary glands [23]. Consider CT of the maxillofacial region when clinically indicated (e.g., worsening mouth opening or suspected ossification), with careful risk–benefit assessment, especially in paediatric patients. If surveillance is needed, low-dose protocols or alternative imaging (e.g., ultrasound) should be prioritised.
- Multidisciplinary approach: We recommend coordination with the rheumatologist and anaesthesiologist before any planned intervention [5,18].

### 4.9. Strengths and Limitations

This study has several notable strengths. To our knowledge, it represents the first systematic, age-stratified characterisation of the dental phenotype in FOP based on a relatively large cohort of genetically confirmed patients (n = 52). The inclusion of three distinct age groups (1–5, 6–17 and 18–35 years) allowed us to capture the progressive nature of oral complications, which has only been suggested in previous case reports and small series. The use of standardised, validated instruments (DMFT index, OHI-S, OHIP-14, calibrated clinical examination with inter-rater reliability assessment) strengthens the reproducibility of our findings. Furthermore, the integration of objective radiological (CT densitometry) and biochemical (sialometry, salivary crystal analysis) measures provides a multidimensional view of FOP-related oral pathology. The correlation between salivary calcium phosphate crystals and CT-documented calcification is a novel finding that opens the possibility of a non-invasive biomarker for disease activity.

Limitations related to sample size and statistical power. A principal limitation of this study is the sample size. Our power calculation indicated that a minimum of 10 patients per group was required; we enrolled 14, 21 and 17, respectively, which only modestly exceeds this threshold. Although this sample is relatively large for an ultra-rare disease like FOP (prevalence < 1 per million), it remains underpowered for subgroup analyses (e.g., comparing rare *ACVR1* variants) and for detecting small-to-moderate effect sizes. The large effect sizes (Cohen’s d > 1.5, Cramér’s V up to 0.72) that we observed increase confidence in the primary comparisons, but the exact prevalence estimates (e.g., 58.8% for enamel hypoplasia) should be interpreted cautiously and require validation in larger, independent cohorts, ideally through international multicentre collaboration.

Several other limitations must be acknowledged. First, the cross-sectional design does not establish causality or true age-related progression; observed differences may reflect cohort effects rather than intra-individual changes. A prospective longitudinal study is needed. Second, although the control group was recruited contemporaneously from the same centres during the same period (2022–2025), it was volunteer-based and may have had better oral health awareness and access to preventive care than the general population, potentially underestimating the true differences between FOP patients and the general population. Additionally, socioeconomic status (SES), fluoride exposure, dental attendance frequency, dietary sugar intake, and oral hygiene habits were not recorded for the control group; these unmeasured confounders are a further limitation. Third, all patients were recruited from three Russian centres; selection bias cannot be ruled out, as patients with milder or more severe forms followed elsewhere may differ. Fourth, survival bias may operate—our cohort includes only alive and consenting patients; those who died early from severe FOP could have a different dental phenotype. Fifth, information bias may arise from self-reported prior invasive procedures (recall bias, especially in older patients). Sixth, detection bias is possible: although examiners were blinded to age group and disease severity, the physical appearance of advanced FOP cannot be completely masked, affecting subjective outcomes like stomatitis grading. Seventh, the sample size (n = 52) is sufficient for primary comparisons but underpowered for subgroup analyses (e.g., rare *ACVR1* variants). Eighth, subjective elements remain in pain assessment and stomatitis grading despite calibration. Ninth, we could not perform histological confirmation of salivary gland calcification because biopsy is contraindicated in FOP. Tenth, generalisability is limited by single-country recruitment, different healthcare access and dietary habits; however, the mutation profile (98.1% *R206H*) is typical worldwide, and the proposed dental phenotype is likely biologically determined. Despite these limitations, the large effect sizes (Cohen’s d > 1.5, Cramér’s V up to 0.72) and the magnitude of differences versus controls (e.g., 84.6% vs. 38.5% for caries) strongly support FOP-specific effects. Eleventh, although we observed a statistically significant correlation between salivary calcium phosphate crystals and CT-based calcification grade (Spearman’s ρ = 0.67, *p* = 0.003), this finding is based on only three patients with detectable crystals (17.6% of Group III); therefore, the correlation coefficient is inherently unstable (95% CI: 0.39–0.84). This finding should be considered hypothesis-generating rather than conclusive. Twelfth, the use of OHIP-14 in Group I (1–5 years) with parent proxy-report, although methodologically unavoidable in pre-school children with rare diseases, lacks formal validation. Consequently, the quality-of-life comparisons between Group I and older groups should be viewed as descriptive rather than definitive. Future studies should develop and validate a disease-specific or age-appropriate proxy-report instrument for young children with FOP.

The generalisability of our findings to other populations requires caution. Our group of patients was exclusively Russian, with universal access to dental care through the state system but potentially different fluoride exposure, dietary sugar intake, and cultural attitudes toward oral hygiene compared to Western European or North American populations. The mutation profile (98.1% *R206H*) is typical for FOP worldwide, so genetic generalisability is high. However, the prevalence of prior invasive dental procedures (82.4% in adults) may reflect local practice patterns and may not be directly applicable to countries where early diagnosis and preventive protocols are already established. The proposed clinical dental phenotype (chronic stomatitis, TMJ dysfunction, enamel hypoplasia) is likely biologically determined and thus generalisable, whereas the exact prevalence estimates (e.g., 58.8% enamel hypoplasia) should be validated in independent cohorts. Our recommendations for non-invasive management (remineralising gels, propofol sedation) are based on pathophysiological principles and can be adopted internationally, but the specific CT monitoring schedule may need adaptation to local resources and radiation safety regulations.

### 4.10. Future Directions

Longitudinal, multicentre studies with larger cohorts are urgently needed to validate the age-related differences we observed [24,25]. Development of a disease specific oral health questionnaire for FOP would improve quality of life assessment. The potential of salivary calcium phosphate crystals as a non-invasive biomarker of disease activity should be tested in prospective studies correlating crystal presence with serum BMP levels and clinical flare up frequency [26,27,28,29,30]. Finally, randomised controlled trials of preventive dental regimens (e.g., CPP ACP gels vs. fluoride alone) are warranted.

## 5. Conclusions

This cross-sectional observational study of 52 genetically confirmed patients with fibrodysplasia ossificans progressiva (FOP) provides the first systematic, age stratified characterisation of the dental phenotype in this ultra-rare disease. Our findings demonstrate that oral complications are not only nearly universal but also follow predictable age-group differences. Chronic stomatitis and temporomandibular joint dysfunction approach 100% prevalence by early adulthood, while enamel hypoplasia, malocclusion and poor oral hygiene accumulate over time, leading to a severe decline in oral health-related quality of life. The observed correlation between salivary calcium phosphate crystals and CT-documented soft tissue calcification—based on only three positive patients—is hypothesis-generating and warrants further prospective validation in larger cohorts before any biomarker claim can be made.

These results have direct clinical implications for dentists, rheumatologists and paediatricians. Based on our cross-sectional observations, we suggest a preliminary clinical dental phenotype of FOP that may aid in early recognition of the disease and in the planning of minimally invasive, preventive dental care. Avoiding iatrogenic trauma—particularly intramuscular injections, extractions and even routine scaling without sedation—is likely to be beneficial to prevent flare-ups and irreversible heterotopic ossification. The practical recommendations derived from our data (use of remineralising gels, non-invasive caries treatment, propofol sedation, and CT monitoring) provide a preliminary framework that may be considered.

Future multicentre, longitudinal studies are needed to validate the age-related trends observed here and to test whether salivary crystal analysis can serve as a real-time marker of disease activity. Until then, our findings suggest that integrating dental care into multidisciplinary management may be beneficial into the multidisciplinary management of FOP from the earliest possible age, with the goal of preserving function and quality of life in a patient population that already faces profound physical limitations.

## Figures and Tables

**Table 1:** Baseline characteristics of the study participants.

Characteristic	Group I (n = 14)	Group II (n = 21)	Group III (n = 17)	*p*-Value ^1^
Age, years, mean (standard deviation (SD))	3.2 (1.4)	11.5 (3.1)	26.4 (5.2)	<0.001
Female, n (%)	8 (57.1)	12 (57.1)	10 (58.8)	0.99
Disease duration, years, mean (SD)	2.1 (1.2)	8.9 (3.4)	18.3 (6.1)	<0.001
*ACVR1 R206H* mutation, n (%)	14 (100)	20 (95.2)	17 (100)	0.54
Prior invasive dental procedures, n (%)	1 (7.1)	8 (38.1)	14 (82.4)	<0.001 ^2^

^1^ *p*-value: Kruskal–Wallis test for continuous variables, chi-square test for categorical variables. ^2^ Chi-square test for linear trend: χ^2^ = 28.4, *p* < 0.001.

**Table 2:** Dental pathologies across age groups.

Indicator	Group I (n = 14)	Group II (n = 21)	Group III (n = 17)	Control Group (n = 156) ^1^	*p*-Value (FDR adj)	Cramér’s V
Caries (DMFT ≥ 4), % (95% CI)	85.7 (12/14) (56.2–98.0)	85.7 (18/21) (63.7–96.9)	82.4 (14/17) (56.6–96.2)	38.5 (60/156) (30.7–46.6)	>0.05	0.07
Chronic stomatitis, % (95% CI)	7.1 (1/14) (0.2–33.9)	38.1 (8/21) (18.1–61.6)	100 (17/17) (80.5–100)	6.4 (10/156) (3.1–11.5)	<0.001	0.72
Enamel hypoplasia, % (95% CI)	21.4 (3/14) (4.7–50.8)	42.9 (9/21) (21.8–66.0)	58.8 (10/17) (32.9–81.6)	4.5 (7/156) (1.8–9.1)	0.03	0.36
Malocclusion (Angle II), % (95% CI)	0 (0/14) (0.0–23.2)	47.6 (10/21) (25.7–70.2)	47.1 (8/17) (23.0–72.2)	14.7 (23/156) (9.6–21.3)	0.008	0.52
OHI-S > 2.0, % (95% CI)	21.4 (3/14) (4.7–50.8)	28.6 (6/21) (11.3–52.2)	29.4 (5/17) (10.3–55.9)	11.5 (18/156) (7.0–17.7)	0.049	0.31

^1^ Control group examined contemporaneously (2022–2025). All comparisons between FOP groups and controls were age-stratified (Group I vs. controls aged 1–5 years, Group II vs. controls aged 6–17 years, Group III vs. controls aged 18–35 years). *p*-values were adjusted using the Benjamini–Hochberg FDR method (q = 0.05). OHI-S = Oral Hygiene Index—Simplified.

**Table 3:** TMJ disorders across age groups.

Type of Disorder	Group I (n = 14)	Group II (n = 21)	Group III (n = 17)	Control Group (n = 156) ^1^	*p*-Value (FDR adj)
Total TMJ disorders, % (95% CI)	7.1 (1/14) (0.2–33.9)	71.4 (15/21) (47.8–88.7)	100 (17/17) (80.5–100)	8.3 (13/156) (4.5–13.8)	<0.001
Limitation of mouth opening (<30 mm), % (95% CI) ^2^	0 (0/14) (0.0–23.2)	57.1 (12/21) (34.0–78.2)	88.2 (15/17) (63.6–98.5)	2.6 (4/156) (0.7–6.5)	<0.001
Painful jaw movement, % (95% CI)	0 (0/14) (0.0–23.2)	33.3 (7/21) (14.6–57.0)	58.8 (10/17) (32.9–81.6)	5.8 (9/156) (2.7–10.7)	<0.001
Crepitation/clicks, % (95% CI)	0 (0/14) (0.0–23.2)	23.8 (5/21) (8.2–47.2)	47.1 (8/17) (23.0–72.2)	3.2 (5/156) (1.0–7.3)	<0.001

^1^ Control values are presented for the corresponding age-stratified subgroups (Group I vs. controls aged 1–5 years, Group II vs. controls aged 6–17 years, Group III vs. controls aged 18–35 years). Maximal mouth opening in adult controls was 41.8 ± 4.5 mm; no control subject had opening <30 mm. *p*-values are FDR-adjusted (Benjamini–Hochberg, q = 0.05). ^2^ Maximal mouth opening in adult controls was 41.8 ± 4.5 mm; no control subject had opening <30 mm.

**Table 4:** OHIP-14 domain scores (mean (95% CI)) ^1^.

Domain	Group I (n = 14)	Group II (n = 21)	Group III (n = 17)	Control (Adults, n = 46) ^2^	*p*-Value ^3^
Physical functioning	78.4 (74.5–82.3)	52.1 (48.8–55.4)	41.3 (38.3–44.3)	84.6 (81.4–87.8)	<0.001
Emotional state	85.0 (82.7–87.3)	63.5 (60.2–66.8)	34.7 (32.2–37.2)	80.3 (77.5–83.1)	<0.001
Social activity	89.2 (86.8–91.6)	70.8 (68.0–73.6)	48.9 (45.5–52.3)	81.5 (78.6–84.4)	<0.001
Total OHIP-14 score	12.4 (10.0–14.8)	34.6 (30.7–38.5)	48.9 (45.4–52.4)	13.8 (11.5–16.1)	<0.001

^1^ Domain scores (physical functioning, emotional state, social activity) are presented as percent of maximum possible quality of life (100% = no impairment, best; 0% = maximum impairment, worst). Total OHIP-14 score is in original metric (0–56, higher = worse). Thus, higher domain scores but lower total OHIP-14 indicate better oral health-related quality of life. ^2^ For controls, OHIP-14 was collected for participants aged ≥ 8 years (n = 132; adult subgroup n = 46). All FOP vs. control differences *p* < 0.001. MID for OHIP-14 total score = 6 points; all differences exceed MID. ^3^ Kruskal–Wallis test; all pairwise differences significant at *p* < 0.01. OHIP-14 = Oral Health Impact Profile-14.

## Data Availability

Our study was conducted under the approval of the Institutional Ethics Committee of Sechenov University (Protocol No. 04-19, dated 6 March 2019). The ethics approval explicitly requires that patient data, even when anonymized, cannot be deposited in public repositories due to strict local data protection regulations and the nature of the informed consent obtained (which did not include public data sharing). Therefore, the data are not publicly available. However, to ensure transparency and reproducibility, the anonymized minimal dataset and the full statistical code (R script) have been prepared as Supplementary Materials. The dataset can be made available to qualified researchers upon reasonable request to the corresponding author, subject to additional approval from the Institutional Ethics Committee.

## Supplementary Materials

The following supporting information can be downloaded at https://www.mdpi.com/article/10.3390/jcm15103951/s1. Statistical analysis code (SPSS syntax version 26.0 and Python boot-strapping script).

## Abbreviations

The following abbreviations are used in this manuscript:

FOP	Fibrodysplasia Ossificans Progressiva
DMFT	Decayed, Missing, Filled Teeth
OHI-S	Oral Hygiene Index—Simplified
TMJ	Temporomandibular Joint
CT	Computed Tomography
FDR	False Discovery Rate
ACVR1	Activin A receptor type I
BMP	Bone Morphogenetic Protein
WHO	World Health Organization
STROBE	Strengthening the Reporting of Observational Studies in Epidemiology
OHStat	Oral Health Statistics (guidelines)
ICC	Intraclass Correlation Coefficient
CI	Confidence Interval
HU	Hounsfield Units
MID	Minimal Important Difference
SD	Standard Deviation
CPP-ACP	Casein Phosphopeptide—Amorphous Calcium Phosphate
