# Supplementary material for: Interdisciplinary Study of the Clinical Phenotype of Patients with Fibrodysplasia Ossificans Progressiva (FOP) in Dental Practice: A Cross-Sectional Clinical–Statistical Analysis
Source: J Clin Med. 2026 May 20;15(10):3951. doi: 10.3390/jcm15103951 (PMC13207639; doi:10.3390/jcm15103951)
Supplement: Supplementary file 1 [file jcm-15-03951-s001.zip › jcm-4312388-supplementary.pdf]

README – Minimal dataset for reproducibility

Statistical analysis code (SPSS syntax version 26.0 and Python bootstrapping script)

No patient level data are porvided.

Due to institutional ethics restrictions (Sechenov University Ethics Committee protocol No. 04-19, 06 March 2019) and patient privacy requirements, the original de-identified dataset cannot be made publicly available. However, the dataset is available from the corresponding author upon reasonable request, subject to approval by the Institutional Ethics Committee.

Variable definitions (for reference):

- subject id: anonymized identifier
- age group: 1 = Group I (1–5 years, deciduous dentition), 2 = Group II (6–17 years, mixed dentition), 3 = Group III (18–35 years, permanent dentition)
- sex: 0 = male, 1 = female
- acvr1_mutation: 1 = R206H, 0 = other
- disease_duration_years: continuous
- prior_invasive_procedures: 0 = no, 1 = yes
- dmft_ge4: 0 = DMFT < 4, 1 = DMFT ≥ 4
- chronic_stomatitis: 0 = absent, 1 = present
- enamel_hypoplasia: 0 = absent, 1 = present
- malocclusion_angle_II: 0 = absent, 1 = present
- ohi_s_gt2: 0 = OHI-S ≤ 2.0, 1 = OHI-S > 2.0
- tmj_total_disorder: 0 = absent, 1 = present
- mouth_opening_mm: continuous (interincisal distance, mm)
- salivary_flow_ml_min: continuous
- salivary_ph: continuous
- salivary_calcium_phosphate_crystals: 0 = absent, 1 = present
- ct_calcification_grade: 0 = none, 1 = mild (<200 HU), 2 = moderate (200–400 HU), 3 = severe (>400 HU)
- ohip14_total_score: continuous (0–56, higher = worse)
- physical_functioning_pct: percent of maximum quality of life (0–100, higher = better)
- emotional_state_pct: percent of maximum quality of life (0–100, higher = better)
- social_activity_pct: percent of maximum quality of life (0–100, higher = better)

How to run the code:

1. The primary analysis was performed using SPSS Statistics version 26.0.
2. SPSS syntax file ("FOP_analysis.sps") is provided.
3. A Python plugin script ("bootstrap_ci_cohen_d.py") for bias-corrected and accelerated bootstrap (1000 replicates) is also included.
4. Place the syntax and script in your working directory.
5. The syntax expects an input file "FOP_data_anonymized.csv". Since this file is not included due to ethics restrictions, the code will produce an error if run without data.
6. To test the code structure, the authors can provide a synthetic (simulated) dataset upon request.

Contact:

For access to the anonymized dataset, please email the corresponding author (aglaya.kazumowa@yandex.ru). Requests will be reviewed by the Institutional Ethics Committee of Sechenov University.
